# Supplementary material for: Statins and Bempedoic Acid: Different Actions of Cholesterol Inhibitors on Macrophage Activation
Source: Int J Mol Sci. 2021 Nov 19;22(22):12480. doi: 10.3390/ijms222212480 (PMC8623589; doi:10.3390/ijms222212480)
Supplement: Supplementary file 1 [file ijms-22-12480-s001.zip › ijms-1437400-supplementary.pdf]

International Journal of
Molecular Sciences

MDPI

Linnenberger et al., 2021

Supplementary Materials

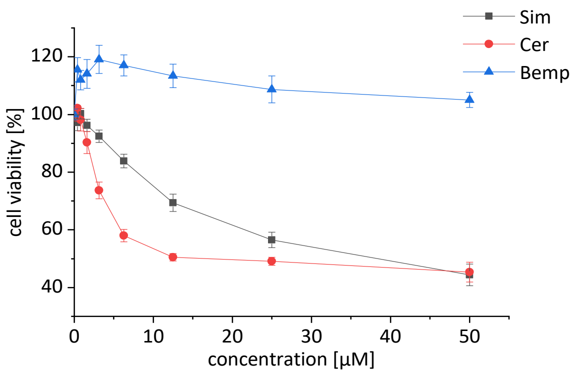

Figure S1. Cytotoxicity of statins and bempedoic acid. BMMs were treated with simvastatin (Sim), cerivastatin (Cer), or bempedoic acid (Bemp) at the indicated concentrations for 24 hours and viability was normalized to the respective DMSO control (Co). Cell viability was measured by MTT (*n* = 3, sixtuplicates).

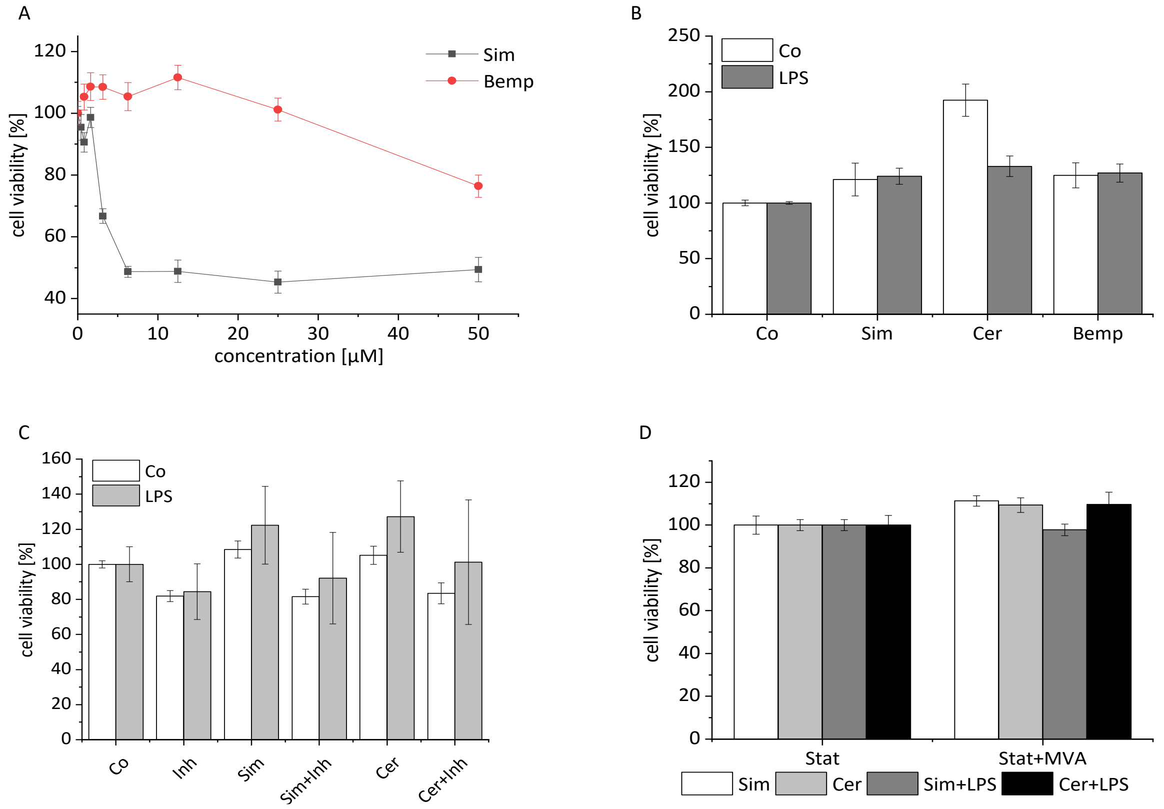

Figure S2. Cytotoxicity of compounds. A. RAW 264.7 cells were treated with simvastatin (Sim), or bempedoic acid (Bemp) at the indicated concentrations for 24 hours and viability was normalized to the respective DMSO control (Co). Cell viability was measured by MTT (n = 3, sixtuplicates). B. RAW-Blue™ cells were stimulated for the last 4 hours with LPS (100 ng/mL) in the presence or absence of simvastatin (Sim, 2 µM), cerivastatin (Cer, 1 µM), or bempedoic acid (Bemp, 25 µM) for 24 hours and viability was normalized to the respective DMSO control. Cell viability was measured by MTT. Co = solvent control (n = 3, triplicates). C. RAW-Blue™ cells were treated with simvastatin (Sim, 2 µM) or cerivastatin (Cer, 1 µM) in the presence or absence of PD98059 (Inh, 10 µM) for 24 h and in the presence or absence of LPS (100 ng/mL) for 4 hours. Cell viability was measured by MTT. Co = solvent control (n = 3, triplicates). D. RAW-Blue™ cells were treated with the statins (Stat) simvastatin (Sim, 2 µM) or cerivastatin (Cer, 1 µM) in the presence or absence of mevalonate (MVA, 100 µM) for 24 h and in the presence or absence of LPS (100 ng/mL) for 4 hours. Cell viability was measured by MTT. Co = solvent control (n = 3, triplicates).

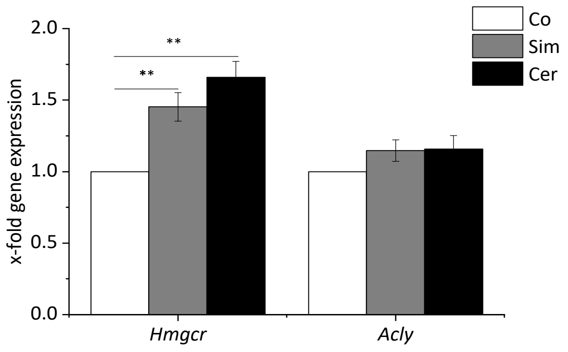

Figure S3. Expression of genes associated with cholesterol synthesis. *Hmgcr* and *Acly* mRNA expression in BMMs were determined by real-time RT-PCR, normalized to *Ppia*, and expressed as x-fold of Co. BMMs were either treated for 24 hours with simvastatin (Sim, 2 μM) or cerivastatin (Cer, 0.5 μM). Co = solvent control. Statistical analysis was performed by one-sample *t*-test followed by Bonholm post hoc test (n = 6). ** $p < 0.01$.

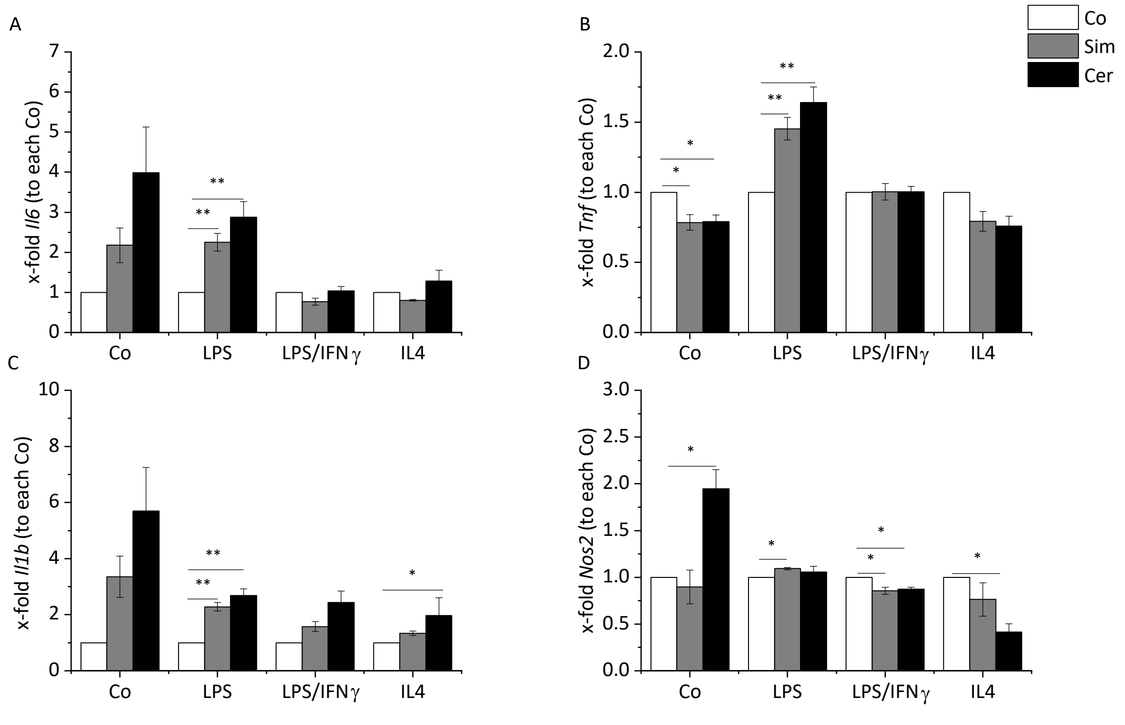

Figure S4. Effect of statin treatment on the inflammatory response in macrophages. A-D. *Il6* (A), *Tnf* (B), *Il1b* (C), and *Nos2* (D) mRNA expression in BMMs were determined by real-time RT-PCR, normalized to *Ppia*, and expressed as x-fold of the respective Co. BMMs were stimulated for the last 4 hours with LPS (100 ng/mL) or polarized towards M1 (LPS, 100 ng/mL; IFNγ, 20 ng/mL), or M2 (IL4, 20 ng/mL) in the presence or absence of simvastatin (Sim, 2 μM) or cerivastatin (Cer, 0.5 μM) for 24 hours. Co = solvent control. Statistical analysis was performed by one-sample *t*-test followed by Bonholm post hoc test for data of the control group. Means of more than two groups were compared by one-way ANOVA with Bonholm post hoc test (normal distribution) (n = 6). * $p < 0.05$, ** $p < 0.01$.

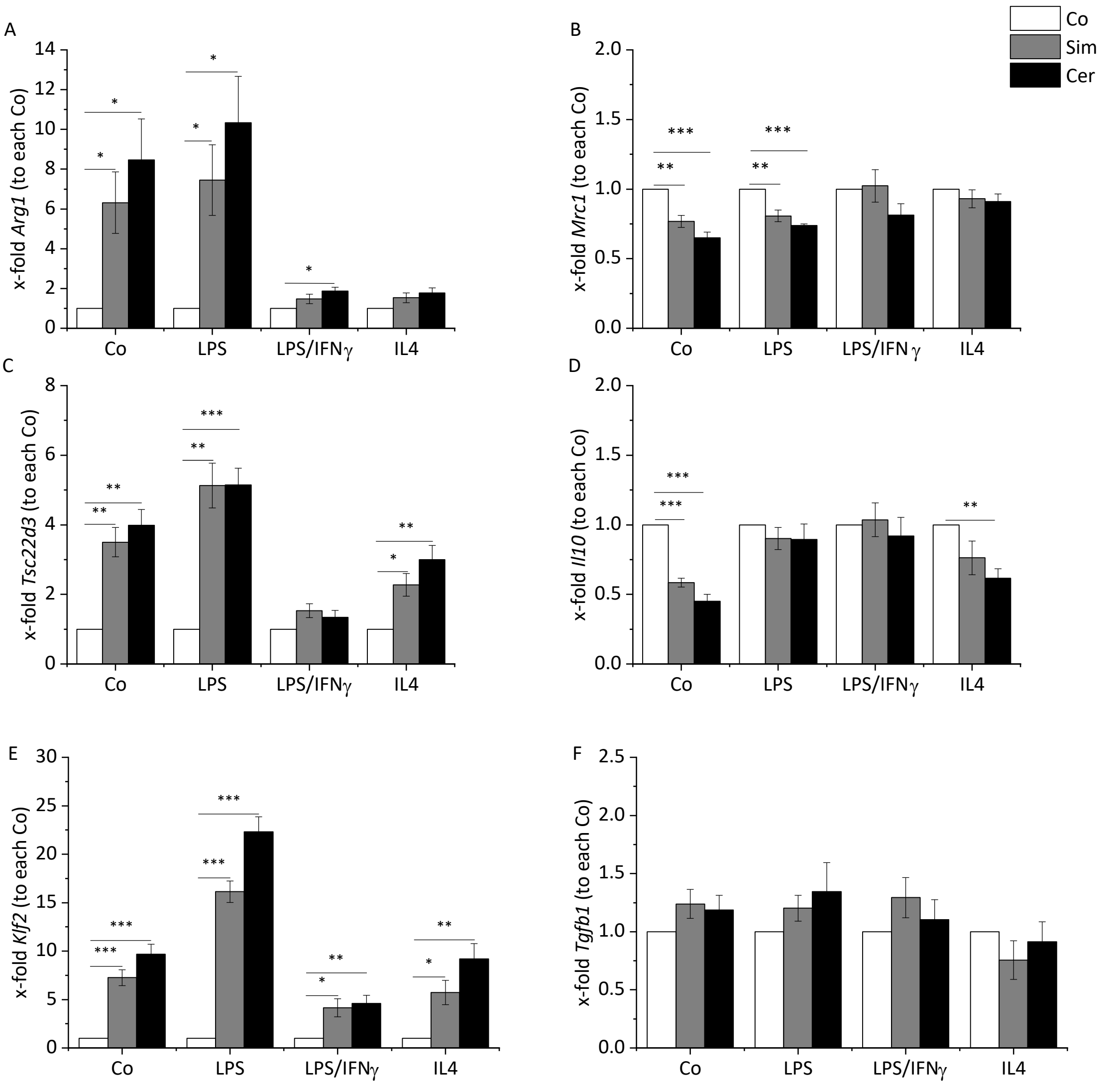

Figure S5. Effect of statin treatment on the anti-inflammatory response in macrophages. A-F. *Arg1* (A), *Mrc1* (B), *Tsc22d3* (C), *Il10* (D), *Tgfb1* (E), and *Klf2* (F) mRNA expression levels in BMMs were determined by real-time RT-PCR, normalized to *Ppia*, and expressed as x-fold of the respective Co. BMMs were stimulated for the last 4 hours with LPS (100 ng/mL) or polarized towards M1 (LPS, 100 ng/mL; IFNγ, 20 ng/mL), or M2 (IL4, 20 ng/mL) in the presence or absence of simvastatin (Sim, 2 μM) or cerivastatin (Cer, 0.5 μM) for 24 hours. Co = solvent control. Statistical analysis was performed by one-sample *t*-test followed by Bonholm post hoc test for data of the control group. Means of more than two groups were compared by one-way ANOVA with Bonholm post hoc test (normal distribution) (n = 6). * $p < 0.05$, ** $p < 0.01$, and *** $p < 0.001$.

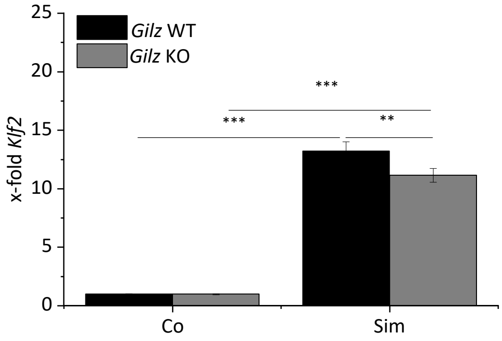

Figure S6. *Klf2* mRNA expression. *Klf2* mRNA expression in BMMs was determined by real-time RT-PCR, normalized to *Ppia*, and expressed as x-fold of Co. BMMs of *Gilz* WT and KO BMMs were treated for 24 hours with simvastatin (Sim, 2 μM). Co = solvent control. Statistical analysis was performed by one-way ANOVA with Bonholm post hoc test (normal distribution) (n = 4, triplicates).**p<0.01, ***p < 0.001.

Control

Phase contrast | Red fluorescence

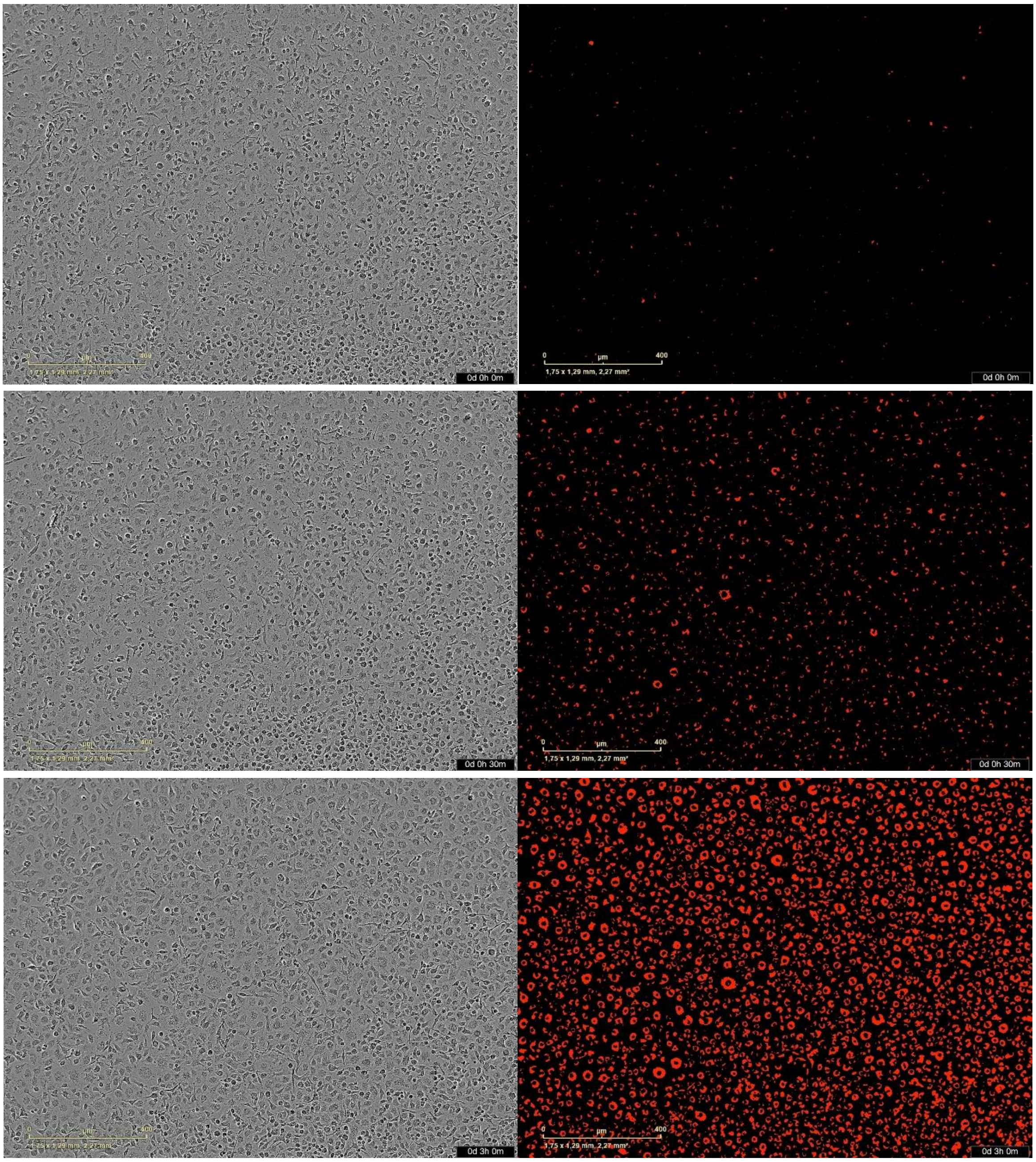

Figure S7. Images shown in Figure 3A (Control) as an overlay are shown as phase contrast and red fluorescence only.

Simvastatin

Phase contrast | Red fluorescence

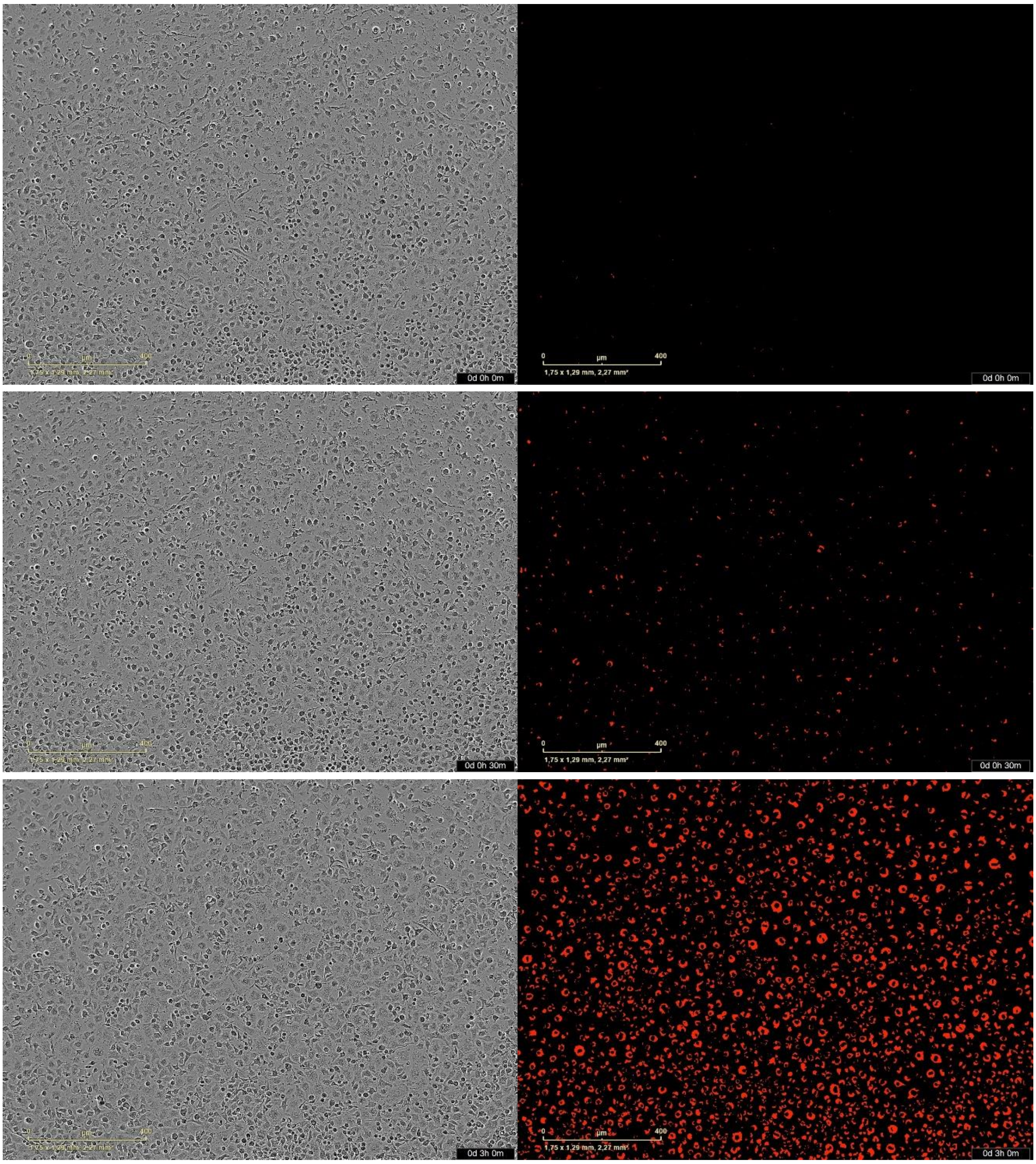

Figure S8. Images shown in Figure 3A (Sim) as an overlay are shown as phase contrast and red fluorescence only.

Cerivastatin

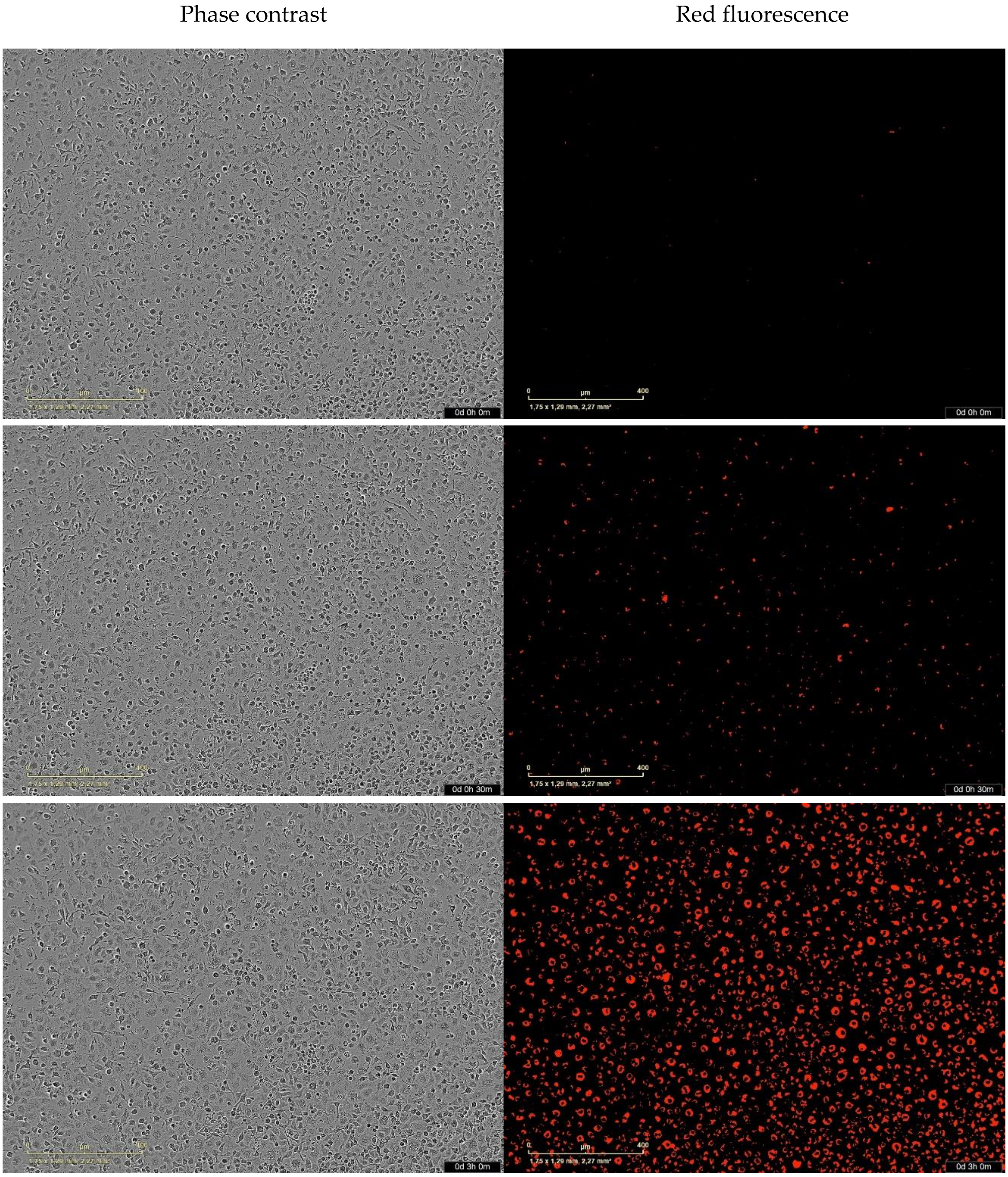

Figure S9. Images shown in Figure 3A (Cer) as an overlay are shown as phase contrast and red fluorescence only.

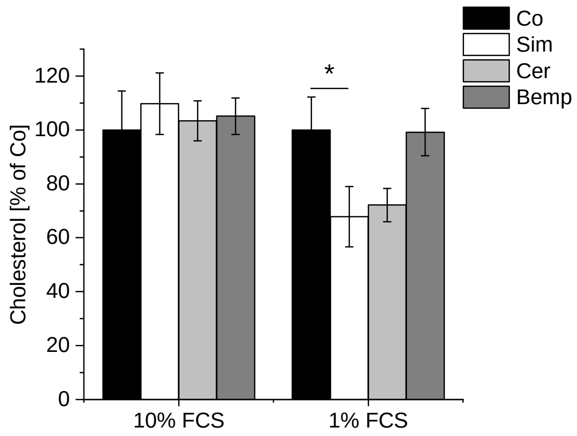

Figure S10. Cellular cholesterol content of statin-treated macrophages under standard and serum-starving conditions. BMMs were either kept in standard medium (10% FCS) or medium containing only 1% FCS and treated for 24 hours with simvastatin (Sim, 2 µM), cerivastatin (Cer, 0.5 µM), or bempedoic acid (25 µM). Intracellular cholesterol levels were assessed using the Amplex assay (n = 2, triplicates). Co = solvent control. Statistical analysis was performed by one-way ANOVA with Bonholm post hoc test (normal distribution). *p < 0.05.

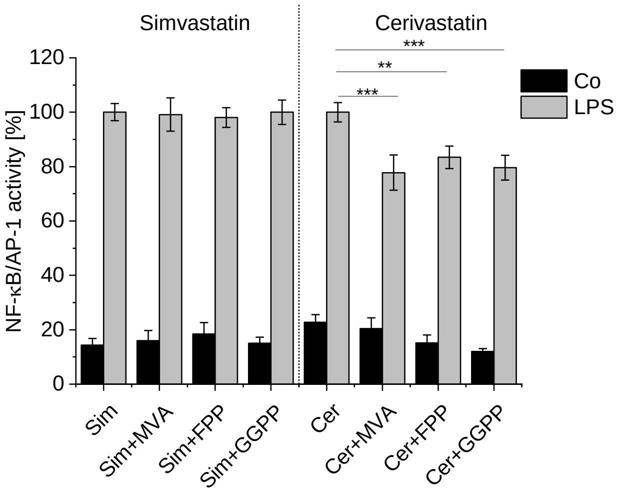

Figure S11. Intermediates of the mevalonate pathway inhibit NF-kb/AP-1-activity in cerivastatin-treated macrophages. RAW-Blue™ cells were treated for 24 hours with either Sim (2 µM) or Cer (1 µM). Cells were co-treated with mevalonate (MVA, 100 µM), farnesyl pyrophosphate (FPP, 10 µM), or geranylgeranyl pyrophosphate (GGPP, 10 µM) where indicated. Inflammatory activation was induced by treatment with LPS (100 ng/mL) for the final 4 hours. NF-κB/AP-1 activity was determined by secreted embryonic alkaline phosphatase (SEAP) detection. Co = solvent control (n = 4, triplicates). Statis-tical analysis was performed by one-way ANOVA with Bonholm post hoc test (normal distribution); **p < 0.01, ***p < 0.001.

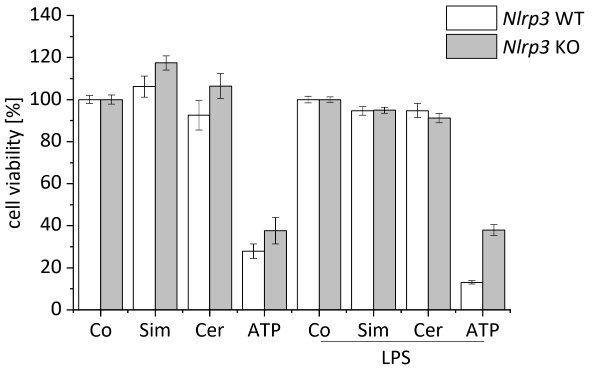

Figure S12. Cytotoxicity of statins in *Nlrp3* WT and KO BMMs. BMMs were treated with simvastatin (Sim, 2 μM), cerivastatin (Cer, 0.5 μM), or ATP as positive control (3 mM, 30 min). Inflammatory activation was induced by treatment with LPS (100 ng/mL) for the final 4 hours. Cell viability was measured by MTT. Co = solvent control (*n* = 4, quadruplicates).

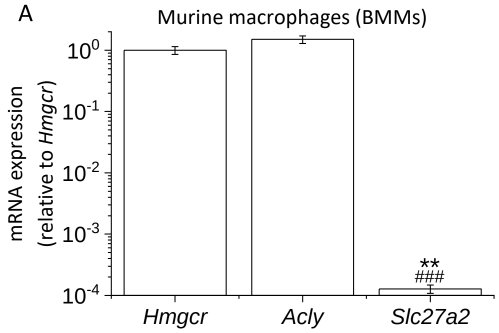

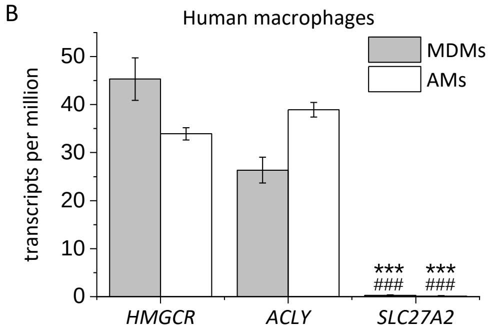

Figure S13. Expression of HMG-CoA reductase (*Hmgcr* / *HMGCR*), ATP-citrate lyase (*Acly* / *ACLY*), and very-long-chain acyl-CoA synthetase-1 (*Slc27a2* / *SLC27A2*) in murine and human macrophages. (A) Gene expression levels in BMMs were determined by real-time RT-PCR, normalized to *Ppia*, and expressed as x-fold of *Hmgcr* (n = 3). (B) Gene expression in human monocyte-derived macrophages (MDMs) and alveolar macrophages (AMs) according to Gene Expression Omnibus (GEO) datasets GSE162669 and GSE162698 (*n* = 3). Statistical analysis was performed by one-way ANOVA with Bonholm post hoc test (normal distribution); ** p < 0.01, *** p < 0.001 *vs. Hmgcr* / *HMGCR*; ### p < 0.001 *vs. Acly* / *ACLY*.

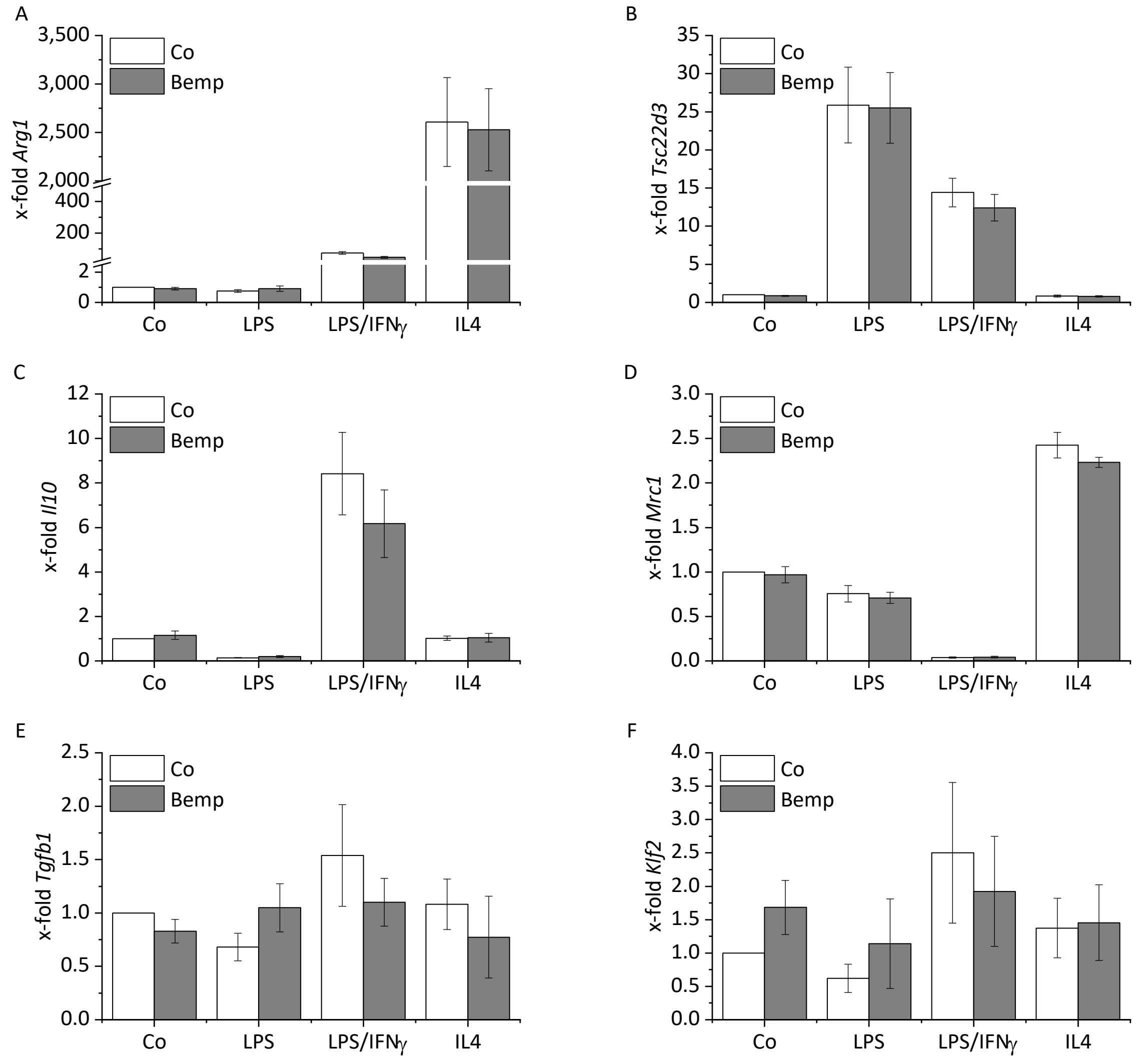

Figure S14. Effect of bempedoic acid treatment on the anti-inflammatory response in macrophages. A-F. *Arg1* (A), *Tsc22d3* (B), *Il10* (C), *Mrc1* (D), *Tgfb1* (E), and *Klf2* (F) mRNA expression in BMMs were determined by real-time RT-PCR, normalized to *Ppia*, and expressed as x-fold of Co. BMMs were stimulated for the last 4 hours with LPS (100 ng/mL) or polarized towards M1 (LPS, 100 ng/mL; IFNγ, 20 ng/mL), or M2 (IL4, 20 ng/mL) in the presence or absence of Bemp (bempedoic acid, 25 μM) for 24 hours. Co = solvent control (n = 6). One-sample t-test followed by Bonholm post hoc test was used for analyzing gene expression data of the control group. Means of more than two groups were compared by one-way ANOVA with Bonholm post hoc test (normal distribution).

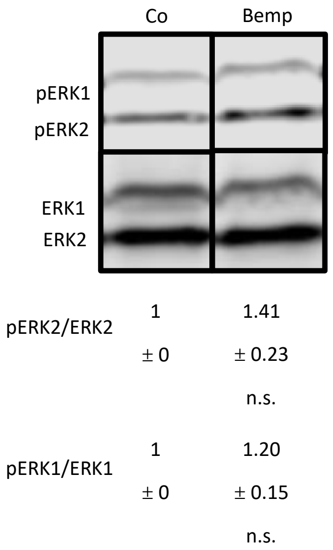

Figure S15. Bempedoic acid does not affect ERK signaling. ERK phosphorylation was examined by Western Blot analysis. BMMs were treated with bempedoic acid (Bemp, 25 µM) for one hour. One representative blot is shown. Signal intensities were quantified and normalized to total ERK and expressed as mean ± SEM. Co = solvent control. Statistical analysis was performed by one-sample *t*-test followed by Bonholm post hoc test. n.s. = not significant (*n* = 3).

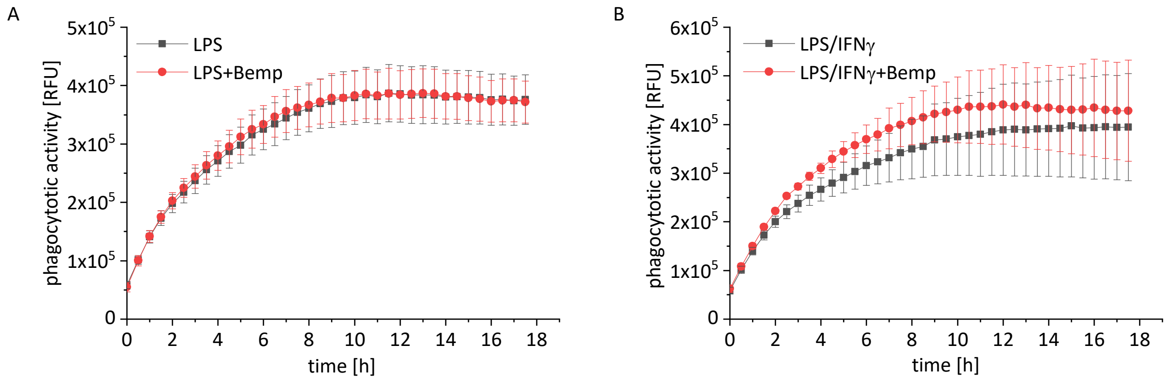

Figure S16. Effect of bempedoic acid treatment on the phagocytotic activity of macrophages. A-B. BMMs were stimulated for the last 4 hours with LPS (100 ng/mL) or polarized towards M1 (LPS, 100 ng/mL; IFNγ, 20 ng/mL) in the presence or absence of bempedoic acid (Bemp, 25 µM) for 24 hours and monitored by an IncuCyte S3 system after addition of fluorogenic pHrodo® Red *S. aureus* bioparticles (5 µg/well). Co = solvent control, RFU = relative fluorescence units. Statistical analysis was performed by two-way ANOVA with Bonholm post hoc test (*n* = 4, duplicates).
